# Host-Microbe-Pathogen Interactions: A Review of *Vibrio cholerae* Pathogenesis in *Drosophila*

**DOI:** 10.3389/fimmu.2019.03128

**Published:** 2020-01-24

**Authors:** Saeideh Davoodi, Edan Foley

**Affiliations:** Department of Medical Microbiology and Immunology, Faculty of Medicine and Dentistry, University of Alberta, Edmonton, AB, Canada

**Keywords:** IMD, *Drosophila melanogaster*, proliferation, *Vibrio cholerae*, insulin, microbiome, metabolism, T6SS

## Abstract

Most animals maintain mutually beneficial symbiotic relationships with their intestinal microbiota. Resident microbes in the gastrointestinal tract breakdown indigestible food, provide essential nutrients, and, act as a barrier against invading microbes, such as the enteric pathogen *Vibrio cholerae*. Over the last decades, our knowledge of *V. cholerae* pathogenesis, colonization, and transmission has increased tremendously. A number of animal models have been used to study how *V. cholerae* interacts with host-derived resources to support gastrointestinal colonization. Here, we review studies on host-microbe interactions and how infection with *V. cholerae* disrupts these interactions, with a focus on contributions from the *Drosophila melanogaster* model. We will discuss studies that highlight the connections between symbiont, host, and *V. cholerae* metabolism; crosstalk between *V. cholerae* and host microbes; and the impact of the host immune system on the lethality of *V. cholerae* infection. These studies suggest that *V. cholerae* modulates host immune-metabolic responses in the fly and improves *Vibrio* fitness through competition with intestinal microbes.

## Introduction

### Background

A complex set of interactions among host intestinal cells, and gut-resident microbes, impacts the viability of all participants. For example, commensal microbes consume intestinal nutrients, and generate metabolites that influence development, growth, metabolism, and immune system function in the host (1–8). Introduction of microbes with pathogenic potential to the gut lumen, or rearrangements to the composition or distribution of gut microbial communities, can have substantial impacts on intestinal homeostasis for the host (9). In particular, shifts in niche occupancy by gut bacteria, or alterations to metabolic outputs from the gut microbiome, can result in the development of severe intestinal disease (10–13). For example, *Bacteroides thetaiotaomicron*, a common human commensal, cleaves host glycans to produce fucose, a sugar that modulates the virulence of enterohemorrhagic *Escherichia coli* (14). Despite the importance of regulated molecular exchanges among host and microbial cells for host fitness and microbial function, our knowledge of pathogen-commensal interactions in the context of immune-metabolic regulation and intestinal disease is still quite limited. To fully understand such complex, multipartite interactions, it is essential that we deploy all relevant experimental systems at our disposal.

*Drosophila melanogaster* is a valuable experimental tool for studying host-microbe interactions. Lab-raised strains of *Drosophila* associate with a limited number of bacterial taxa (15–17), dominated by easily cultivated *Acetobacter* and *Lactobacillus* strains that are accessible to genetic manipulation, and deployment in large-scale screens. Researchers have access to simple protocols for the establishment of flies with a defined intestinal microbiome (18, 19), and there is an abundance of publicly available lines for the genetic manipulation of fly intestinal function. Combined, these advantages allowed researchers to make substantial breakthroughs in understanding how flies interact with intestinal bacteria (20). Importantly, given the extent to which genetic regulators of intestinal homeostasis are conserved between vertebrates and invertebrates (20, 21), discoveries made with the fly have the potential to illuminate foundational aspects of host-microbe interactions. However, there are several key differences to note between flies and vertebrates that partially limit the utility of the fly model. Specifically, flies lack lymphocyte-based adaptive defenses, and the fly microbiome is considerably different to that reported in vertebrates.

### Antimicrobial Defenses in the Fly Intestine

**Figure 1:**
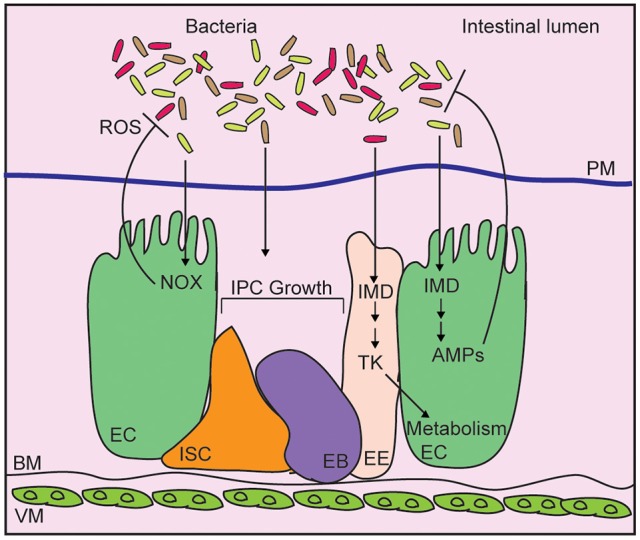
Schematic representation of the adult *Drosophila* midgut. Intestinal bacteria are contained within the lumen by a chitinous peritrophic matrix (PM). Bacteria diaminopimelic acid-type peptidoglycan activates the immune deficiency (IMD) pathway in enterocytes (EC), leading to production of antimicrobial peptides (AMP). In enteroendocrine cells (EE), IMD controls expression of the metabolism-regulatory hormone Tachykinin (Tk). Epithelial reactive oxygen species (ROS) generated by NADPH oxidases (NOX) also contribute to bacterial killing while cues from the bacterial microbiome promote the growth of intestinal progenitor cells (IPC), composes of intestinal stem cells (ISC), and enteroblasts (EB).

*Drosophila* integrate physical, chemical, proliferative, and antibacterial strategies to neutralize intestinal microbes, and prevent systemic infection of the host (Figure 1) (22, 23). The chitinous peritrophic matrix lines the midgut, and presents a physical barrier against bacterial invasion (24), similar to the mucus lining of the vertebrate intestinal tract. The germline-encoded immune deficiency (IMD) antibacterial defense pathway, a signaling pathway similar to the mammalian Tumor Necrosis Factor pathway (25), detects bacterial diaminopimelic acid-type peptidoglycan, and acts through the NF-κB transcription factor family member, Relish, to induce expression of antimicrobial peptides (26–29). At the same time, Dual Oxidase (Duox) and NADPH Oxidase (Nox) protect the host from gut bacteria through the generation of bactericidal reactive oxygen species (30, 31). Evolutionarily conserved growth regulatory pathways respond to damage of epithelial cells by promoting a compensatory growth and differentiation of intestinal stem cells (ISCs) in infected flies (32–35). This adaptive repair mechanism maintains the epithelial barrier, and prevents systemic infection of the host. Combined, these antibacterial defenses protect the host from infection, and maintain beneficial relationships between the fly and their gut microbiome.

### The *Drosophila* Microbiome

The fly microbiome is transmitted horizontally through the deposition of bacteria on the outer surface of freshly laid embryos, and is maintained through the ingestion of food contaminated with bacteria (36). Gut bacteria regulate *Drosophila* intestinal homeostasis by affecting metabolism, growth, and immunity in the host. Interactions between the host and gut microbiota have been extensively covered in several recent reviews (20, 37–39), and will not be discussed in detail here. In brief, detailed studies have uncovered roles for symbiotic *Lactobacillus* and *Acetobacter* species in the control of fly metabolism and growth (40–42). For example, a dehydrogenase activity in *Acetobacter pomorum*, produces acetic acid that regulates insulin signaling, carbohydrate, and lipid levels in the host (40). In addition to the effects of individual symbionts on nutrient allocations in the host, interactions among bacterial communities have significant effects on host metabolism, growth, and physiology (43–47). *Vibrio cholerae* (*V. cholerae*) has emerged as a particularly useful tool to study interactions between the host, the intestinal microbiota, and an enteric pathogen. A pioneering study in 2005 established that flies are susceptible to oral infection with *V. cholerae*, dying within a few day from a diarrheal disease with symptoms similar to cholera in humans (48). The genetic tractability of the fly and *V. cholerae* established this system as a very attractive model to identify key host and microbial determinants of pathogenesis. In the following years, a number of studies uncovered complex roles for metabolism, host immunity, epithelial growth, and microbial antagonism in the outcome of *V. cholerae* pathogenesis in the fly. In this review, we will discuss key findings from these studies, and outline what they tell us about host-microbe interactions in general, and *V. cholerae*-mediated pathogenesis in particular.

### *Vibrio cholerae*: Pandemics and Pathogenicity

*Vibrio cholerae* is a curved, Gram-negative member of the *Vibrionaceae* family of Proteobacteria (49). It inhabits aquatic environments, and copepods and chironomids are reported as natural reservoirs in marine ecosystems (50, 51). Intestinal colonization by *V. cholerae* causes the diarrheal disease, cholera, and is considered a substantial public health threat, especially in countries with poor sanitation and contaminated water (52). The first cholera pandemic emerged in 1817, with an expansion of cholera beyond the Indian subcontinent (53). Since then, the world has witnessed an additional six pandemics, with the seventh pandemic ongoing (54). Models that estimate cholera burden predict ~3 million cases of disease per year, resulting in roughly 100,000 deaths (55).

*Vibrio cholerae* strains are divided into classical and non-classical serotypes, with classical ones expressing the O1 antigen on their surface (56, 57). Classical serotypes are further subdivided into two biotypes—classical and El Tor—that differ in the expression of a number of markers, such as hemolysins (58–61). The outbreak of epidemic cholera that spread through southeast Asia in 1992 is caused by the non-classical strain of *V. cholerae* 0139 (62), whereas the ongoing pandemic that originated in Indonesia in 1961 is caused by the El Tor biotype (63). El Tor causes a milder cholera disease (64), with infected individuals frequently remaining asymptomatic early in infection (65).

*Vibrio cholerae* encodes several virulence factors that regulate survival, colonization, and pathogenicity (66–69). Cholera toxin (CT) is a hexameric adenosine diphosphate-ribosyl transferase that contains one A subunit surrounded by five B subunits (70, 71). Upon release into the intestinal lumen via a type two secretion system (72), the B pentamer of CT interacts with host GM1 ganglosides (73), permitting toxin endocytosis, and a subsequent cytosolic release of the A1 subunit (74). A1 ADP-ribosylates the Gs alpha subunit, locking Gα_*s*_ in an active state (75). Active Gα_*s*_ elevates adenylate cyclase activity, greatly increasing levels of 3′,5′-cyclic AMP, resulting in excess protein kinase A (PKA) activity (76). PKA stimulates an efflux of chloride ions through the cystic fibrosis transmembrane conductance regulator channel (77), leading to an uncontrolled flow of water, sodium and potassium ions into the intestinal lumen. This extreme, and rapid, dehydration results in the voluminous rice-water diarrhea that hallmarks cholera disease (78). In addition to CT, *V. cholerae* require the toxin co-regulated pilus virulence factor for pathogenesis (79). Toxin co-regulated pilus is a type IV pilus system that mediates colonization of the small intestine by a self-associate mechanism that supports the formation of bacterial microcolonies (80). Toxin co-regulated pilus also serves as the receptor for the CTXφ bacteriophage. CTXφ encodes *ctxAB*, and converts benign *V. cholerae* to pathogenic strains. The ability to synthesize toxin co-regulated pilus is advantageous for *V. cholerae* in aquatic environments, as it improves *V. cholerae* fitness by facilitating inter-bacterial interactions during colonization of host chitinaceous surfaces (81).

Although fluid replacement through oral rehydration solutions, antibiotic therapy, and vaccines are effective treatment options for patients with cholera, increased rates of antibiotic resistance among classical (82) and non-classical (83) strain of *V. cholerae* complicate treatment of the disease. Therefore, new antibacterial strategies that effectively target *V. cholerae* virulence factors are critical to contain this deadly disease. Over the last century, a variety of animal models that include rabbits, mice, fish, and flies, have been used to study *Vibrio*-host interactions and each of these models have added to our understanding of virulence, host responses to infection, interactions between *Vibrio* and host microbes, and cholera vaccine development.

### The Rabbit Model

The first animal model to study *V. cholerae* dates back to 1884 when Nicati and Rietschin inoculated *V. cholerae* into the duodenum of guinea pigs, resulting in cholera-like symptoms (84). Since then, both infant and adult rabbit models of cholera have been widely used by researchers (85–87). As adult rabbits are resistant to oral infection with *V. cholerae*, the pathogen is typically introduced to the animal by ligated ileal loop surgery. In this technique, the small intestine of the rabbit is sealed at two ends, and the pathogen is delivered by injection into the ligated loop, allowing direct measurement of intestinal fluid secretion (88). The rabbit model has been very instructive for understanding *V. cholerae* distribution in the small intestine during infection, the importance of the mucosal barrier to prevent systemic infection of *V. cholerae*, and mechanisms of *V. cholerae* attachment to the intestinal epithelium (89, 90). As infant rabbits are capable of developing toxin co-regulated pilus-dependent cholera, they have been useful to study the reactogenicity associated with developing live attenuated *V. cholerae* vaccines as well (91). However, despite these advances, *Vibrio* pathogenesis studies using the rabbit ligated ileal loop model are labor-intensive, and do not replicate the normal route of infection. An alternative, oro-gastric infection model with infant rabbits pre-treated with the stomach acid production inhibitor, Cimetidine, allows oral infection and provides a valuable adult mammal model that circumvents needs for surgical interventions (87).

### The Mouse Model

The infant or suckling mouse in commonly used to study *V. cholerae* pathogenesis (92). In this model, infant mice are infected via the oro-gastric route. In the infant mouse, the intestinal microbiome has not fully developed, allowing *V. cholerae* to colonize the host with diminished colonization resistance from commensal microbes. Studies working with infant mice have uncovered essential virulence factors of *V. cholerae*. For example, the toxin co-regulated pilus (93), and ToxR (69), which regulates toxin co-regulated pilus expression were originally characterized in the suckling mouse model. The adult mouse model was also a significant contributor to understanding the mechanisms of *V. cholerae* pathogenesis using accessory toxins such as hemolysin, hemagglutinin/protease, and multifunctional auto-processing RTX toxin (94). These observations were important to understand the ability of *V. cholerae* to express toxins other than CT to prolong its colonization in the host without severe diarrheal symptoms. However, this model comes with some limitations, as suckling mice do not develop watery diarrhea, and lymphocyte-based immune defenses are not fully developed in the host (95–97). Furthermore, as infant mice are separated from their mothers, they have a limited survival and reduced timeframe for research performance. Adult mice are less efficient for cholera studies as they are naturally resistant to *V. cholerae* colonization (98). Thus, manipulations such as removal of intestinal microbes by antibiotic treatment (99), or infection by ligated ileal loop surgery (100), are necessary for colonization of adult mice with *V. cholerae*.

### The Zebrafish Model

*V. cholerae* is found in the intestinal tract of fish in the wild, where the bacteria degrades macromolecules ingested by fish via its chitinase and protease, building a commensal relationship between fish and *V. cholerae* (101). Analysis of cholera patients from an outbreak in 1997 showed that dried fish consumption was significantly associated with the spread of disease, implicating fish as potential vector for *V. cholerae* (102). Building on associations between fish and *V. cholerae* in the wild, the zebrafish, *Danio rerio*, has recently been developed recently as a natural host model to study *V. cholerae* (103). Importantly, pathogenic strains of *V. cholerae* cause a cholera-like disease characterized by host intestinal colonization, epithelial destruction, diarrhea, and the expulsion of live pathogens (103). Unlike the adult rabbit model, researchers do not require surgical interventions prior to infection, and in contrast to the mouse model, investigators are not restricted to working with antibiotic-treated juveniles (103, 104). Fish and humans have similarly complex microbiomes that shift with age and diet (105), making fish a useful model to study interactions between commensal bacteria and the invading pathogen (106). However, it is important to note that fish cannot be raised in axenic conditions, and it is technically challenging to generate and maintain fish populations with fully defined microbiota for sustained periods.

### The *Drosophila* Model

Insects such as chironmids (107) and houseflies (108) are candidate reservoirs of *V. cholerae*, and some studies suggest a correlation between disease transmission and increases in fly population, during cholera outbreaks, or in areas where the disease is endemic (109). Given the association of *V. cholerae* with arthropod vectors, researchers tested the utility of *Drosophila* as a model to characterize *V. cholerae* pathogenesis. *Drosophila* infections typically involve oral delivery of the pathogen, or introduction of the pathogen into the body cavity of the fly through a septic injury (110). In contrast to non-pathogenic *Vibrio* strains, injection of *V. cholerae* into the body cavity resulted in a rapid death of infected flies, raising the possibility of using flies as a model to study *V. cholerae* pathogenesis (111). In a foundational study from 2005, researchers showed that continuous feeding of adult flies with *V. cholerae* caused a cholera-like disease characterized by loss of weight, and rapid death that required a functional Gα_*s*_ in the host (48), establishing flies as a valuable model to characterize *V. cholerae* pathogenesis. However, in contrast to vertebrates, *ctx* mutants remain lethal to flies, suggesting CT-independent pathogenic mechanisms in adult flies. Furthermore, *Vibrio* polysaccharide-dependent biofilm formation is important for persistent colonization of the fly rectum and for *V. cholerae*-mediated lethality (112), whereas *Vibrio* polysaccharides interfere with colonization of the host intestine (113). Thus, the fly is a useful tool to identify uncharacterized virulence factors that affect interactions between *V. cholerae* and an arthropod host. As studies with this model progress, it will be interesting to determine how such virulence factors impact pathogenesis in vertebrate models.

### *Vibrio cholerae* and the IMD Pathway

The IMD pathway modifies expression of host genes that control processes as diverse as bacterial killing, metabolism, and intestinal homeostasis (114–121). Mutations in the IMD pathway are linked with intestinal phenotypes that implicate IMD as a critical modifier of host-bacteria interactions. For example, IMD is required to survive enteric infections with entemopathogenic *Pseudomonas entomophila* (122). Additionally, IMD pathway mutants are characterized by changes to the composition of the intestinal microbiome, modified distribution of live bacteria throughout the intestine (123), and elevated bacterial loads in the intestine (17, 123–127). It is tempting to speculate that IMD controls bacterial populations through the direct release of antimicrobial peptides into the gut lumen. This hypothesis is supported by a recent study that confirmed a failure to contain infectious Gram-negative and fungal pathogens in flies that lack antimicrobial peptide genes (29). However, we cannot exclude the possibility that IMD-dependent control of bacterial populations includes inputs from other processes such as intestinal metabolism. Consistent with this hypothesis, studies have revealed links between immune and insulin activity in several models (128–132), including flies (120, 133–138), and IMD activity controls expression of the metabolism-regulatory peptide, Tachykinin, in enteroendocrine cells of the anterior midgut (117). In addition to metabolic deregulation, IMD pathway mutants are characterized by accelerated proliferation of intestinal progenitor cells, intestinal tissue dysplasia, and early death (34). Many of these phenotypes are reverted by elimination of the gut microbiome (124), confirming links between IMD, gut microbial composition, and intestinal health. As flies are highly amenable to modifications of intestinal gene activity, *Drosophila* has emerged as a particularly valuable tools to characterize links between host epithelial immunity, and *V. cholerae* pathogenesis.

In flies, reactive oxygen species generation does not appear to affect *V. cholerae* pathogenesis (139). In contrast, septic injury of adult flies with *V. cholerae* causes elevated expression of IMD-responsive antimicrobial peptides. Furthermore, induced expression of antimicrobial peptide genes attenuated *V. cholerae* pathogenesis in the septic injury model (111). These observations suggest that IMD will have protective effects against *V. cholerae*. However, characterization of flies challenged with *V. cholerae* through the natural, oral route, revealed an unexpected link between host immunity and pathogenesis. Specifically, although oral infection promotes the expression of IMD-responsive antimicrobial peptides in the intestine, IMD pathway mutants displayed an enhanced survival after oral infection with *V. cholerae* (140), indicating that host immune activity contributes to *V. cholerae* pathogenesis. Follow-up work showed that mutations in the IMD pathway have minimal effects on levels of intestinal *V. cholerae* (139). Nonetheless, whereas *V. cholerae* inhibit ISC growth in wild-type flies, ISC proliferation is unimpaired in the intestines of *V. cholerae*-infected IMD pathway mutants (139) suggesting that *V. cholerae*-dependent activation of IMD inhibits ISC proliferation, accelerating host death.

Studies of links between host immunity and *V. cholerae* pathogenesis uncovered an involvement of the *Drosophila* oxidation resistance 1 ortholog*, mustard* (*mtd*), in host viability (139, 141). Mustard is a Lysine Motif domain-bearing protein with roles in pupal eclosion (142). A gain-of-function mutant, *mtd*^*EY*04695^, that increases expression of a nuclear localized mustard isoform, significantly improves the survival duration of flies infected with *V. cholerae* (141). Molecular work showed that *mtd*^*EY*04695^ mutants process the IMD-responsive NF- κB transcription factor Relish normally, and express most antimicrobial peptides to wild type levels after infection (139, 141). However, genome-wide transcriptional studies uncovered broad overlaps between the expression profiles of *mtd*^*EY*04695^ and an IMD pathway mutant, including diminished expression of the *diptericin* antimicrobial peptide, suggesting interactions between mustard function and IMD activity. Similar to IMD pathway mutants, *mtd*^*EY*04695^ flies are capable of progenitor cell growth after infection, supporting the notion of links between immune activity, ISC proliferation, and host survival. Looking forward, it will be interesting to characterize the immune phenotypes of loss-of-function mutations in the *mtd* locus.

A recent study from our group examined the consequences of IMD inactivation in defined intestinal cell types for host viability after infection with *V. cholerae* (143). We found that inhibition of IMD in differentiated enterocytes significantly extended the survival times of infected flies, whereas inhibition of IMD in the progenitor cell compartment shortened survival times. These observations suggest that the activity of IMD in enterocytes is sufficient to enhance *V. cholerae* pathogenesis. The mechanism by which immune activity influences *V. cholerae* pathogenesis requires clarification. In this context, we note that IMD is required for the delamination of damaged cells in the intestinal epithelium (119). As *V. cholerae* causes extensive damage to the midgut epithelium (139, 140, 144), we consider it is possible that *V. cholerae* kills the host, in part, by activating IMD-dependent sloughing of the epithelium. In this untested model, excess delamination effectively disrupts the epithelial barrier, preventing the transduction of growth cues to progenitor cells, and leading to systemic infection and host death. However, we cannot exclude alternative, and potentially non-exclusive mechanistic links, such as metabolic dysfunction, between immune activity and host mortality. In particular, there is a considerable amount of data linking intestinal metabolism to disease progression in infected flies.

### *Vibrio cholerae* and Host Metabolism

The gut microbiota modifies metabolism in *Drosophila*, with implications for host growth and development (40, 42, 145). For example, symbiotic *Ap* are a source of thiamine during development (146). Additionally, *Ap*-derived acetate stimulates insulin signaling activity in the fly (40). The *Drosophila* insulin response pathway is highly similar to the vertebrate counterpart (147), and *Ap*-dependent control of insulin activity affects key developmental processes such as intestinal growth, size regulation, and storage of energy (40). Similar to *Ap*, symbiotic *Lactobacillus plantarum* plays an important role in the regulation of larval growth. In this case, *Lp* activates intestinal peptidases, at least partially in an IMD-dependent manner (148), to promote the uptake of amino acids from the larval growth medium, thereby activating the Target of Rapamycin complex, and promoting larval growth (42). When considering microbial control of host metabolism, it is important to note that higher-order interactions in a complex community of intestinal bacteria impact host health and fitness (43). For example, interactions between symbiotic *Acetobacter* and *Lactobacillus* species influence lipid homeostasis in adult flies (149).

A genetic screen for *V. cholerae* mutants with impaired pathogenesis in flies identified the CrbRS two-component system as a modifier of host killing (150). CrbRS is composed of the CrbS histidine kinase sensor, and the CrbR response regulator. CrbRS controls expression of *acetyl CoA-synthase (acs1)*, a bacterial regulator of acetate consumption. In *E. coli*, expression of *acs1* activates the acetate switch, whereby bacteria switch from production to consumption of the short-chain fatty acid, acetate (151). The acetate switch is conserved in *V. cholerae*, as mutations in *crbR, crbS*, or *acs1* prevent consumption of acetate by *V. cholerae* in liquid culture (150, 152). These observations suggest that *V. cholerae*-dependent virulence may involve consumption of intestinal acetate by the pathogen. Consistent with that hypothesis, provision of dietary acetate was sufficient to extend survival times in flies infected with *V. cholerae*. Mechanistically, the authors showed that consumption of intestinal acetate by wild-type *V. cholerae* disrupted insulin signaling in the host, leading to intestinal steatosis and depletion of lipid stores from the fly fat body, an insect organ with functional similarities to the vertebrate liver and white adipose tissue (153). Removal of lipids from the fly medium prevented steatosis, and extended host viability, confirming a role for lipid homeostasis in *V. cholerae* pathogenesis. Interestingly, CrbS is expressed during *V. cholerae* infections in mice and humans (154, 155), raising the possibility that pathogenic consumption of intestinal acetate is a general virulence strategy of *V. cholerae*.

Links between metabolism and pathogenesis extend beyond short-chain fatty acid consumption. For example, mutations of the *V. cholerae* glycine cleavage system also attenuate virulence in the fly model (156). These mutants colonize fly intestines with equal efficiency as wild-type *V. cholerae*, indicating that the phenotype is likely a consequence of an increased ability of the host to tolerate infection. In line with this hypothesis, glycine cleavage mutants fail to suppress ISC division, and do not affect lipid levels in fat tissue or homeostasis. Instead, glycine cleavage mutants have increased levels of methionine-sulfoxide in their intestines, and dietary supplementation with methionine-sulfoxide, or mutation of the host Methionine sulfoxide reductase A (MsrA) gene extended host viability and restored lipid homeostasis to flies infected with *V. cholerae*, implicating methionine-sulfoxide availability in pathogenesis.

Metabolic regulation is also sensitive to quorum-sensing by *V. cholerae*. A recent study showed that quorum sensing in the El tor C6706 strain minimizes pathogenesis in flies, as deletion of the quorum-sensing master regulator, *hapR*, increased pathogenesis (157). HapR suppresses the expression of CT (158), and toxin co-regulated pilus virulence factors (159), and inhibits expression of *Vibrio* polysaccharide (160, 161), a biofilm exopolysaccharide that enables colonization of the *Drosophila* rectum (112). The elevated pathogenesis observed in Δ*hapR* strains was not the result of increased biofilm formation. Instead, the phenotype appears to be the consequence of increased succinate uptake by Δ*hapR* due to elevated expression of the *Vibrio cholerae* INDY succinate transporter. Consistent with this model, supplementation of the infection medium with succinate significantly extended survival times of flies infected with Δ*hapR*. Similar to phenotypes associated with methionine-sulfoxide, and acetate, succinate consumption by *V. cholerae* was associated with depletion of lipid stores from the fat body, suggesting a possible role for inter-organ regulation of lipid homeostasis in the survival of infection with *V. cholerae*.

### *Vibrio cholerae* and ISC Growth

Much of the data above describe the phenotypic impacts of *V. cholerae*-mediated consumption of intestinal metabolites. However, it is important to remember that *V. cholerae* competes with gut-resident bacteria for attachment to the intestinal niche (162). Thus, *V. cholerae*-dependent displacement of intestinal bacteria can also affect the profile of metabolites available to the host. For example, *V. cholerae* encodes a type six secretion system (T6SS) that delivers an array of toxins to susceptible prokaryotic, and eukaryotic, prey (163–165). Two studies from our group implicated the T6SS in *Drosophila* pathogenesis mediated by the El Tor strain, C6706. The first study showed that the T6SS targets symbiotic *Acetobacter pasteurianus* for killing, and that the T6SS contributes to host killing (144). T6SS-dependent killing of the host requires the presence of *Ap*, and association of adult flies exclusively with T6SS-refractory *Lactobacillus* species is sufficient to extend the viability of C6706-infected hosts. These data indicate that T6SS-mediated killing of flies proceeds through an indirect route that requires host association with *Acetobacter*.

More recently, we showed that the T6SS also affects epithelial renewal in infected flies. In agreement with previous work (139), we showed that *V. cholerae* causes extensive damage to the midgut epithelium, but fails to activate compensatory proliferation in basal progenitor cells (166). Removal of the T6SS diminishes epithelial damage, and restores renewal in infected midguts. These effects are not the result of direct interactions between the T6SS and the host epithelium, as removal of the intestinal microbiome restores renewal capacity to midguts infected with C6706. Collectively, these data indicate that the T6SS contributes to *V. cholerae*-mediated inhibition of epithelial renewal in a manner that requires a gut microbiome. In these assays, inhibition of renewal is not a simple consequence of interactions between *V. cholerae* and symbiotic *Acetobacter*. Instead, inhibition of renewal required association of infected flies with a tripartite community of gut bacteria, consisting of *Ap, Lactobacillus brevis*, and *Lp*, suggesting that T6SS-dependent arrest of progenitor growth is the result of complex interactions between the pathogen and a community of symbionts. Interestingly, quorum sensing appears to be an important factor in progenitor renewal. In vertebrates, the master quorum sensor regulator, *hapR* is not expressed at early stages of infection, where *V. cholerae* are present in low density. The absence of HapR allows for production of the toxin coregulated pilus, and CT, resulting in disease. As *V. cholerae* numbers increase, quorum sensing-dependent production of HapR results in a repression of virulence genes. In our studies, we used a C6706 strain with low *hapR* expression (167), allowing for expression of virulence genes in the fly. In contrast, earlier studies with several C6706 strains that express *hapR* failed to arrest progenitor growth, and were not pathogenic to flies (157). Mutation of *hapR* in these strains restored pathogenesis, and blocked proliferation. In total, these studies hint at a sophisticated interplay between quorum sensing, bacterial competition, and epithelial renewal in the host. It will be interesting to determine the mechanistic basis for these interactions in future studies.

## Conclusion and Future Direction

**Figure 2:**
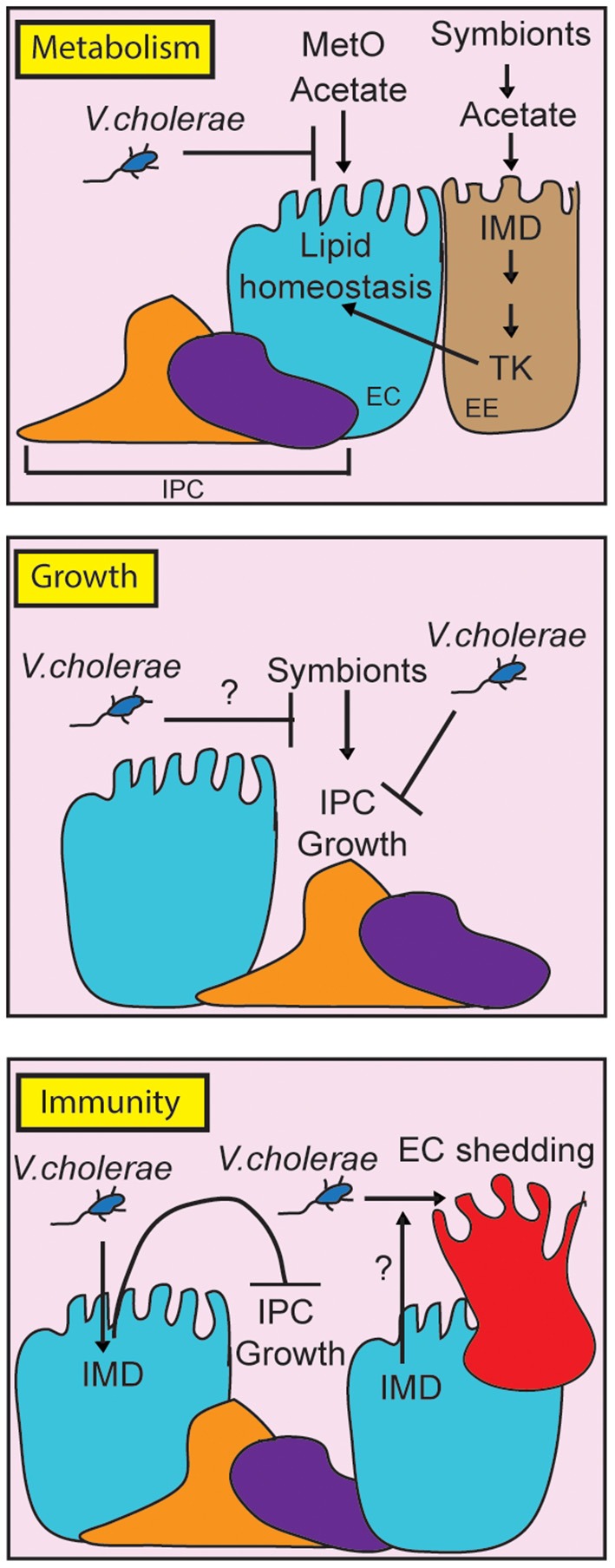
Schematic representation of the impact of pathogenic *Vibrio cholerae* on metabolism, growth and immunity in the adult *Drosophila* midgut. For clarity, we have broken the individual responses into separate panels, although it is important to note that growth, metabolism and immunity share regulatory components *in vivo*. By consuming metabolites such as methionine sulfoxide (MetO) and acetate, *V. cholerae* affects lipid homeostasis contributing to death. At the same time, *V. cholerae* impairs IPC growth pathways, although it is unclear how this affects symbiont-dependent growth responses (indicated with a question mark). Finally, the host IMD pathway contributes to pathogenesis by impairing IPC growth, and possibly by affecting epithelial turnover (indicated by a question mark).

In this review, we have discussed the utility of *D. melanogaster* as an experimental model to understand *V. cholerae* pathogenesis. In the last 15 years, work with the fly uncovered a complex series of interactions between the invading pathogen, the intestinal microbiome, and host defense mechanisms (Figure 2). *V. cholerae* disrupts lipid metabolism in enterocytes, and in the fat body, suggesting impacts of the pathogen on communication between these critical regulators of lipid homeostasis. Host immune defenses contribute to pathogenesis, as IMD pathway mutants survive infections longer than their wild-type counterparts, and display an improved epithelial renewal response. It will be interesting to determine the mechanistic links between immune activity and epithelial renewal, and to determine how changes to lipid metabolism impact pathogenesis. We also consider it important to remember that growth, immunity, and metabolism share numerous regulatory components. The fly is a particularly valuable model to ask how these evolutionary conserved pathways interact to orchestrate systemic responses to a global health threat.

## Author Contributions

SD and EF wrote the paper.

### Conflict of Interest

The authors declare that the research was conducted in the absence of any commercial or financial relationships that could be construed as a potential conflict of interest.
